# 1363. Preliminary Safety Results from a Phase 1 First in Human Study of VYD222: an Extended Half-Life Monoclonal Antibody (mAb) in Development for COVID-19 Prevention

**DOI:** 10.1093/ofid/ofad500.1200

**Published:** 2023-11-27

**Authors:** Kathryn Mahoney, Deepali Gupta, Yong Li, Natalia Betancourt, Aanika Das, Ed Campanaro, Pete Schmidt

**Affiliations:** Invivyd, Inc., Waltham, Massachusetts; Invivyd, Inc., Waltham, Massachusetts; Invivyd, Inc., Waltham, Massachusetts; Invivyd, Inc., Waltham, Massachusetts; Invivyd, Inc., Waltham, Massachusetts; Invivyd, Inc., Waltham, Massachusetts; Invivyd, Inc., Waltham, Massachusetts

## Abstract

**Background:**

VYD222 is a fully human IgG1 mAb demonstrating broad and potent in vitro neutralizing activity across SARS-CoV-2 variants including XBB.1.5. VYD222 is a re-engineered version of adintrevimab, an Fc-modified mAb that has a robust safety data package and demonstrated clinically meaningful results in Phase 2/3 clinical trials for both treatment and prevention of COVID-19 (NCT04805671, NCT04859517). VYD222 is currently in development for COVID-19 prevention.

**Methods:**

This is an ongoing Phase 1, first in human, blinded, randomized, placebo (PBO)-controlled, single ascending dose study of VYD222 administered as slow intravenous (IV) push to healthy adults aged 18-65 years (NCT05791318). Participants were randomized 8:2 in each of 3 cohorts (n=8 VYD222, n=2 PBO): VYD222 1500 mg, 2500 mg, and 4500 mg. Safety and tolerability will be assessed as incidence of treatment emergent adverse events, including serious adverse events (SAEs). Serum pharmacokinetic (PK) parameters will be calculated for VYD222 using noncompartmental analysis methods. Serum virus neutralizing antibody (sVNA) titers of VYD222 ex vivo against relevant SARS-CoV-2 variants will be assessed using a validated assay. Safety, tolerability, PK, and sVNA titers will be assessed through 12 months post-dose. Here we report preliminary blinded safety results from cohorts 1 and 2.

**Results:**

Overall, 12 participants received VYD222 or PBO. Through a minimum of 14 days post dose, among 10 participants in cohort 1 (VYD222 1500 mg n=8, PBO n=2), there were 9 AEs (adverse events) reported, all of which were mild in severity and considered unrelated to study drug. No participant experienced SAEs, infusion-related reactions, or hypersensitivity reactions. Through 2 days post dose, 2 sentinel participants in cohort 2 (VYD222 2500 mg n=1, PBO n=1) experienced zero AEs, study drug related AEs, SAEs, infusion-related reactions, or hypersensitivity reactions. Enrollment continues in cohort 2 as of the data cutoff (04/25/2023) for this abstract. Data remain blinded currently.

**Conclusion:**

To date, a single dose of VYD222, up to 2500 mg IV push, was well tolerated across all healthy participants receiving either VYD222 or PBO in this ongoing phase 1 study. These findings support continued clinical development of VYD222.

**Disclosures:**

**Kathryn Mahoney, PharmD**, Invivyd, Inc.: Employee|Invivyd, Inc.: Stocks/Bonds **Deepali Gupta, BSc**, Invivyd, Inc.: Employee|Invivyd, Inc.: Stocks/Bonds **Yong Li, PhD**, Invivyd, Inc.: Employee|Invivyd, Inc.: Stocks/Bonds **Natalia Betancourt, MS**, Invivyd, Inc.: Employee|Invivyd, Inc.: Stocks/Bonds **Aanika Das, PharmD**, Invivyd, Inc.: Employee|Invivyd, Inc.: Stocks/Bonds **Ed Campanaro, MS**, Invivyd, Inc.: Employee|Invivyd, Inc.: Stocks/Bonds **Pete Schmidt, MD**, Invivyd, Inc.: Employee|Invivyd, Inc.: Stocks/Bonds

